# Histological transformation into pulmonary sarcomatoid carcinoma from lung adenosquamous carcinoma after radical resection of EGFR gene Exon-21 L858R mutation: a case report and literature review

**DOI:** 10.3389/fonc.2026.1811812

**Published:** 2026-06-18

**Authors:** Daxia Cai, Jun Li, Qiyi Chen, Xingdong Cai, Feng Tian, Yanyan Zhu, Jingjing Cao, Jianhui Huang, Xiu Lan, Zhifeng Tian, Jianfei Tu, Yonghui Wang

**Affiliations:** 1 Cancer Center, Lishui Central Hospital, The Fifth Affiliated Hospital of Wenzhou Medical University, Lishui Hospital of Zhejiang University, Lishui, Zhejiang, China; 2 Department of Pulmonary and Critical Care Medicine, The First Affiliated Hospital of Jinan University, Guangzhou, China; 3 Department of General Surgery, Taihe Hospital, Hubei University of Medicine, Shiyan, Hubei, China; 4 Department of General Surgery, Lishui People’s Hospital, the Six Affiliated Hospital of Wenzhou Medical University, Lishui, Zhejiang, China; 5 Department of Pharmacy, Lishui Municipal Central Hospital, The Fifth Affiliated Hospital of Wenzhou Medical University, Lishui, Zhejiang, China; 6 Pathology department, The Fifth Affiliated Hospital of Wenzhou Medical University, Lishui Hospital of Zhejiang University, Lishui, Zhejiang, China

**Keywords:** EGFR-TKIs, histological transformation, non-small cell lung cancer (NSCLC), pulmonary sarcomatoid carcinoma, resistance mechanism

## Abstract

Pulmonary sarcomatoid carcinoma (PSC) is frequently underdiagnosed or misdiagnosed due to its rarity and complex histological features. To date, no universally recommended treatment regimens have been established for this rare subtype, and clinical management is currently extrapolated primarily from standard non-small cell lung cancer (NSCLC) protocols. Herein, we report a case of PSC initially diagnosed as multiple primary lung cancer, including adenosquamous carcinoma and adenocarcinoma (pT1N0M0, IA stage, EGFR Exon-21 L858R mutation, below the detection limit) and adenocarcinoma (pT2aN0M0, stage IB, EGFR Exon-21 L858R mutation). Despite radical surgical resection followed by targeted therapy with afatinib (30 mg orally once daily), the disease recurred. Repeat biopsy confirmed the diagnosis of PSC secondary to histological transformation. The patient achieved a durable complete response (CR) to combined immunochemotherapy. We further review the existing literature on EGFR-TKI-associated histological transformation in lung cancer, to provide guidance for the clinical management of this challenging clinical scenario.

## Introduction

The 2021 WHO classification revised this taxonomy, reclassifying sarcomatoid carcinomas into three separate disease entities: pleomorphic carcinoma, pulmonary blastoma, and carcinosarcoma. Of note, giant cell carcinoma and spindle cell carcinoma were redefined as morphological variants of pleomorphic carcinoma in the updated classification (1). Pulmonary sarcomatoid carcinoma (PSC) is an extremely rare and aggressive subtype of non-small cell lung cancer (NSCLC), characterized by epithelial-mesenchymal transition (EMT), poor prognosis, and resistance to conventional chemotherapy and radiotherapy (2, 3). PSC is an epithelial-derived malignancy. Its sarcomatoid components arise from epithelial carcinoma cells that undergo epithelial-mesenchymal transition (EMT), leading to the loss of epithelial phenotypes (e.g., basement membrane attachment) and the acquisition of high-grade mesenchymal features including enhanced migratory and invasive capacity, apoptosis resistance, and extracellular matrix degradation ability. Pathologically, epithelial carcinoma components are characterized by expression of epithelial biomarkers such as pan-cytokeratin (AE1/AE3) and thyroid transcription factor-1 (TTF-1), while the sarcomatoid components typically express mesenchymal biomarkers including vimentin (4–6). Recurrent genomic alterations identified in PSC include TP53, EGFR (7), KRAS, MET, and ALK fusions, among others (8).

To date, no consensus guidelines have been established for adjuvant chemotherapy, palliative chemotherapy, immunotherapy, or targeted therapy in PSC management (9). The inferior survival outcomes of PSC are primarily attributed to its poor response to conventional systemic therapies, including intrinsic resistance to standard chemotherapy and low responsiveness to radiotherapy, as well as a high recurrence rate after surgical resection (10). Notably, most PSC tumors exhibit high PD-L1 expression and an immune-inflamed tumor microenvironment, which underpins their favorable response to immune checkpoint inhibitors (ICIs) (11). However, to date, no large-scale clinical trials have investigated ICI plus chemotherapy regimens specifically in PD-L1-positive PSC populations.

Herein, we report a case of early-stage multiple primary mixed adenosquamous lung carcinoma harboring EGFR exon 21 L858R mutation, which recurred after radical surgery and underwent histological transformation to PSC following EGFR-TKI treatment with afatinib. We describe the efficacy and safety profile of sintilimab combined with chemotherapy in this patient with advanced PD-L1-high, TP53-mutant PSC, who achieved a durable complete response. We further discuss the role of EGFR-TKIs in driving histopathological transformation in NSCLC, to provide real-world evidence for this rare clinical scenario.

## Case presentation

**Figure 1:**
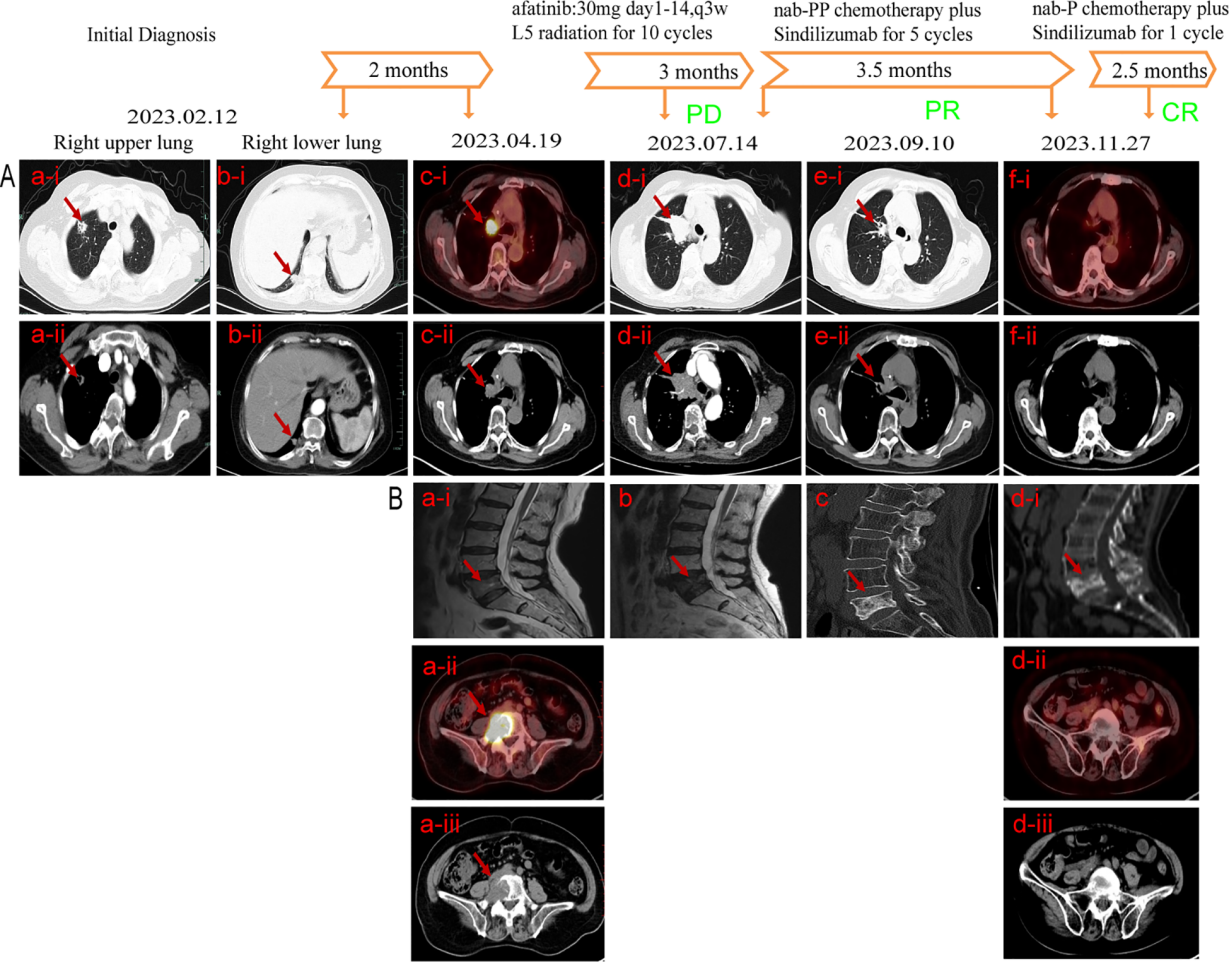
Chest PET/CT/Lumbar MRI/CT images at different time points. **(A)** Chest CT/PET showing intrapulmonary lesions. [**(A)**, a] Preoperative chest CT showing the lesion in the right upper lung lobe. [**(A)**, b] Preoperative chest CT showing the lesion in the right lower lung lobe. [**(A)**, c] PET-CT on April 19, 2023 showing postoperative recurrence of the right upper lung lesion. [**(A)**, d] Chest CT on July 14, 2023 showing the status of the right upper lung lesion. [**(A)**, e] Chest CT on September 10, 2023 showing the status of the right upper lung lesion. [**(A)**, f] PET-CT on November 27, 2023 showing the status of the right upper lung lesion. **(B)** CT/PET-CT/MRI showing the metastatic lesions of the L5 vertebral body. [**(B)**, a-i] MRI on April 19, 2023 showing the L5 vertebral metastatic lesion. [**(B)**, a-ii, a-iii] PET-CT on April 19, 2023 showing the L5 vertebral metastatic lesion. [**(B)**, b] MRI on July 14, 2023 showing the L5 vertebral metastatic lesion. [**(B)**, c] CT on September 10, 2023 showing the L5 vertebral metastatic lesion. [**(B)**, d-i] CT on November 27, 2023 showing the L5 vertebral metastatic lesion. [**(B)**, d-ii, d-iii] PET-CT showing the L5 vertebral metastatic lesion (November 27, 2023). CT, Computed tomography; MRI, magnetic resonance imaging; PET, positron emission tomography; PD, Progressive Disease; PR, Partial Response; CR, Complete Response.

In February 2023, a 68-year-old female presented to our hospital with a 1-month history of persistent cough and expectoration. Chest computed tomography (CT) revealed a heterogeneous lesion in the segment of the left lower lobe measuring approximately 1.8 cm in maximum diameter. The lesion exhibited an internal cavitation, and its margin showed features such as fingerlike projections and long hairs. There was some slight pulling of the adjacent pleura. The CT value of the lesion was around 20 Hounsfield Unit (HU) on the enhanced scan, while the CT value of the lesion was around 65HU on the contrast scan (**Figure 1A**(a). A second, similar solid nodule measuring 15 × 12 mm was detected in the right upper lobe (**Figure 1A**(b)). The patient had no history of smoking or family history of lung cancer. Palpation of superficial lymph nodes was unremarkable, and no evidence of distant metastasis was identified on initial staging workup. The blood biochemical examination was normal, and the tumor markers carcinoembryonic antigen (CEA), Squamous Cell Carcinoma Antigen (SCCA), cytokeratin 19 fragments, neuron-specific enolase, and pepsinogen precursor release peptide were within the normal range. The Cancer Antigen 125 (CA125) level was 50.7 U/ml (reference range <35 U/ml), and the ferritin level was 325 ng/ml (reference range 4.6–204 ng/ml).

After comprehensive clinical evaluation, the patient was diagnosed with synchronous early-stage primary lung cancer: the right upper lobe lesion was staged as cT1N0M0, stage IA, and the right lower lobe lesion as cT2aN0M0, stage IB, with both lesions considered technically resectable for potential cure. Following a multidisciplinary team (MDT) discussion, the patient underwent bilateral video-assisted thoracoscopic surgery (VATS) under general anesthesia with endotracheal intubation on February 14, 2023. The surgical procedure included wedge resection of the right upper lobe, wedge resection of the right lower lobe, mediastinal lymph node sampling, and pulmonary adhesiolysis. Intraoperatively, linear adhesions were noted between the right posterior parietal pleura and the chest wall; no pleural effusion was observed, and the intercostal spaces were well developed. The right upper lobe lesion, measuring 2.0 × 2.0 × 1.5 cm, was identified with overlying pleural dimpling; the right lower lobe lesion, measuring 0.8 × 1.0 × 1.0 cm, also presented with associated pleural indentation. Nodes from stations 4, 7, 10, and 11 were slightly enlarged, with soft texture and dark discoloration, and were sampled for histopathological examination.

**Figure 2:**
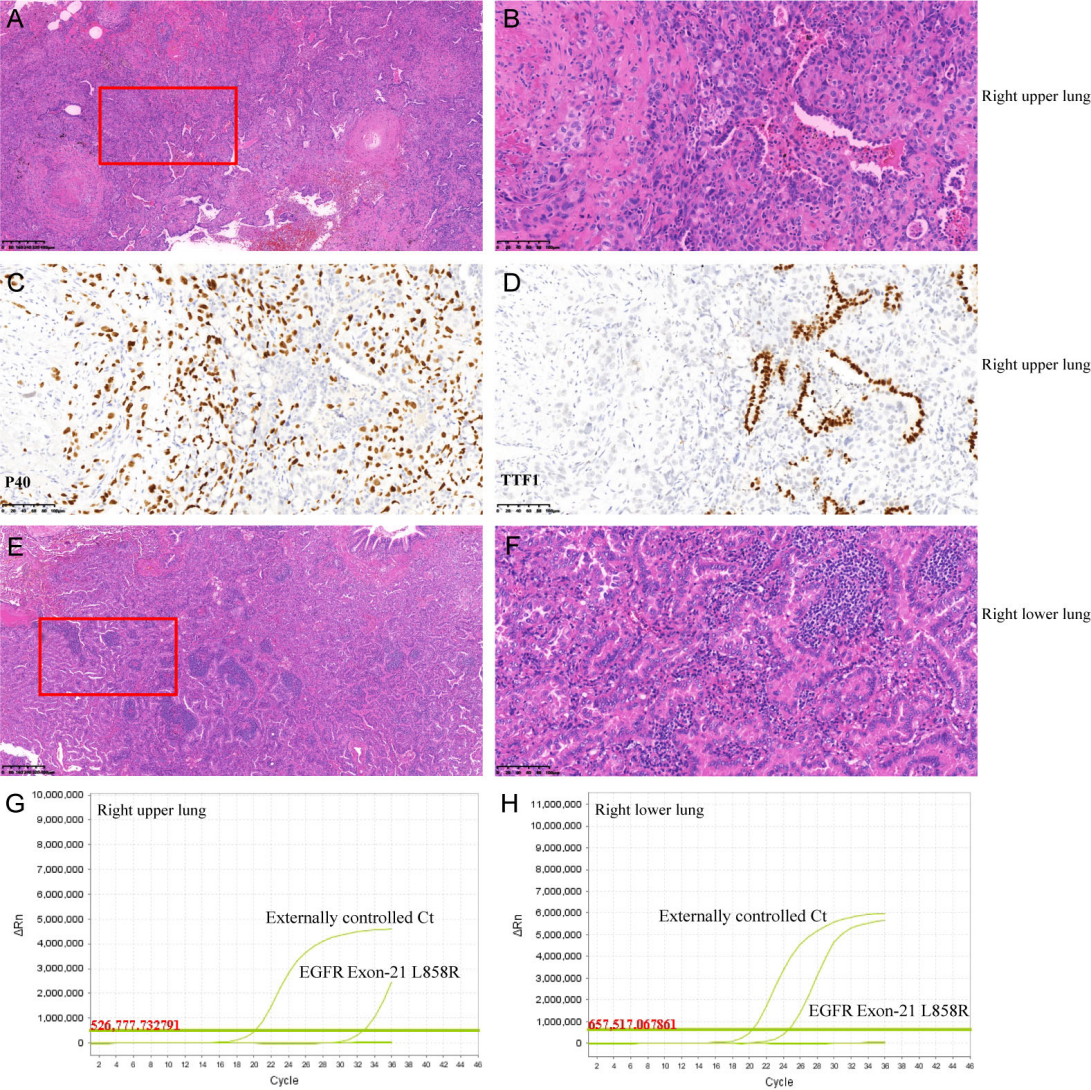
The histopathology, immunohistochemistry (IHC), and genetic testing of the patient’s surgical excision specimen. **(A, B)** H&E stain in Right upper lung, **(A)** original magnification ×40, **(B)** original magnification ×200. **(C, D)** Immunohistochemistry in Right upper lung, original magnification ×200, **(C)** IHC P40, **(D)** IHC TTF1. **(E, F)** H&E stain in Right lower lung, **(E)** original magnification ×40, **(F)**original magnification ×200. **(G, H)** The EGFR/ALK/ROS1 gene mutation RNA detection by fluorescence quantitative, **(G)** EGFR L858R mutation detection result of right upper lung lesion (EGFR gene Exon-21 L858R mutation, but lower than the detection standard’s lower limit, Ct = 30), **(H)** EGFR L858R mutation detection result of right lower lung lesion (positive, Ct = 22). **(G, H)** shows the amplification curves of the FAM channel. The judgment criteria for FAM signal (positive determination standard) are as follows: If the Ct value of the mutation-specific amplification is <31, the sample is directly determined as positive; if 31 ≤ the mutation Ct value <34 and the ΔCt value (Ct value of internal control minus Ct value of the mutation) < 11, the sample is also determined as positive.

Histopathological examination of the right upper lobe surgical specimen obtained on February 24, 2023, confirmed the diagnosis of adenosquamous carcinoma, composed of two distinct histological components: approximately 80% moderately to poorly differentiated adenocarcinoma and 20% poorly differentiated squamous cell carcinoma. On low-power hematoxylin and eosin (H&E) staining, the adenocarcinoma component exhibited a predominant acinar architecture, interspersed with solid trabecular or sheet-like growth patterns; foci of the two components were either admixed or spatially separated, as shown in **Figure 2A**. On high-power H&E staining, the adenocarcinoma cells were columnar or cuboidal, with marked nuclear pleomorphism, arranged in tubular, reticular, or solid patterns (**Figure 2B**). The squamous cell carcinoma component demonstrated a trabecular or sheet-like growth pattern, with tumor cells arranged in clusters, small nests, or cohesive sheets (**Figure 2B**).

Immunohistochemical (IHC) profiling validated the lineage specificity of the two histological components, consistent with the morphological diagnosis of adenosquamous carcinoma: P40 (ΔNp63, a squamous lineage-specific transcription factor) was absent in the adenocarcinoma compartment, while showing strong, diffuse nuclear immunoreactivity in the squamous cell carcinoma component (**Figure 2C**). TTF-1(a canonical pulmonary adenocarcinoma lineage marker) exhibited diffuse nuclear positivity in the adenocarcinoma component, with complete loss of expression in the squamous cell carcinoma compartment (**Figure 2D**). In addition, we performed multi-level deep sectioning on the surgically resected specimen of the right upper lung nodule for immunohistochemical detection, including Vimentin immunohistochemistry. The detailed immunohistochemical results are presented in the **Supplementary Figure** of **Supplementary Materials**. The immunohistochemical findings of the surgically resected right upper lung nodule specimen are shown below: In adenocarcinoma component, EMA, AE1/AE3, TTF-1, NapsinA and CK7 are positive, and P40, CK5/6, Vimentin, CgA and Syn are negative. In squamous cell carcinoma component, EMA, AE1/AE3, P40 and CK5/6 are positive, and TTF-1, NapsinA, Vimentin, CgA and Syn are negative. Additionally, Ki-67-positive cells accounted for 80% of the adeno-squamous carcinoma, which can assess the rapid proliferation rate of tumor cells. The EGFR/ALK/ROS1 gene mutation test indicates a L858R mutation in the EGFR gene’s Exon-21, which is lower than the detection standard’s lower limit (**Figure 2G**). Additionally, the PD-L1 expression levels for both histological components were negative. According to the size of the tumor and the number of lymph node metastases, we re-classified each histopathological component of the disease as follows: pulmonary squamous cell carcinoma was classified as pT1N0M0, IA stage; pulmonary adenocarcinoma was classified as pT1N0M0, stage IA.

The histological section of the right lower lung tissue indicated an invasive adenocarcinoma (measuring 1.5 × 1.0 × 0.6 cm) (**Figures 2E, F**). Under light microscopy, H&E staining revealed that under low magnification, cells appeared as an acidic structure, while under high magnification, they were observed to be columnar or cubical with significant heterogeneity, predominantly arranged in an acinar structure (**Figure 2F**). And EGFR/ALK/ROS1 gene mutations were detected, and EGFR gene Exon-21 L858R mutation was identified (**Figure 2H**). The PD-L1 expression has not been detected. After disease reassessment, the pathology components were classified as pT2aN0M0, stage IB.

She was advised to undergo adjuvant targeted therapy, but she refused. Unfortunately, due to recurrent back pain for one week, she was hospitalized again two weeks after surgery. Lumbar magnetic resonance imaging (MRI) on April 17, 2023 revealed compression and flattening of the L5 vertebral body. Nodular and patchy abnormal signals were observed in the L5 vertebral body and its right posterior element, presenting hypointensity on T1-weighted imaging (T1WI), hyperintensity on T2-weighted imaging (T2WI) and fat-suppressed sequences, with marked heterogeneous enhancement on contrast-enhanced scan, which was indicative of L5 vertebral body metastasis (**Figure 1B**(a-i)). On April 19th, 2023, Positron Emission Tomography/Computed Tomography (PET/CT) scan indicated a recurrence in the right upper lobe of the lung and L5 vertebral body metastasis (**Figure 1A**(c), **Figure 1B**(a-ii), **Figure 1B**(a-iii). We selected 2 measurable lesions as target lesions for efficacy evaluation: ①Recurrent lesion in the right upper lung: Baseline PET-CT on April 19, 2023 showed an irregular nodule adjacent to the main bronchus of the right upper lobe, with lobulation and spiculation at the margin, measuring approximately 1.9 × 2.2 cm on lung window, with abnormally elevated uptake and a maximum SUV of 15.0 (**Figure 1A**(c). This lesion meets the measurable lesion criteria of RECIST 1.1, with baseline scan slice thickness ≤ 5 mm, conforming to the imaging evaluation specifications (12). ②L5 vertebral body metastasis: Baseline PET-CT on April 19, 2023 showed osteolytic destruction of the L5 vertebral body and right posterior element, with local soft tissue mass formation, abnormally elevated uptake and a maximum SUV of 16.1 (**Figure 1B**(a-ii), **Figure 1B**(a-iii). This lesion meets the measurable lesion criteria of PERCIST 1.0, conforming to the imaging evaluation specifications (13).

**Figure 3:**
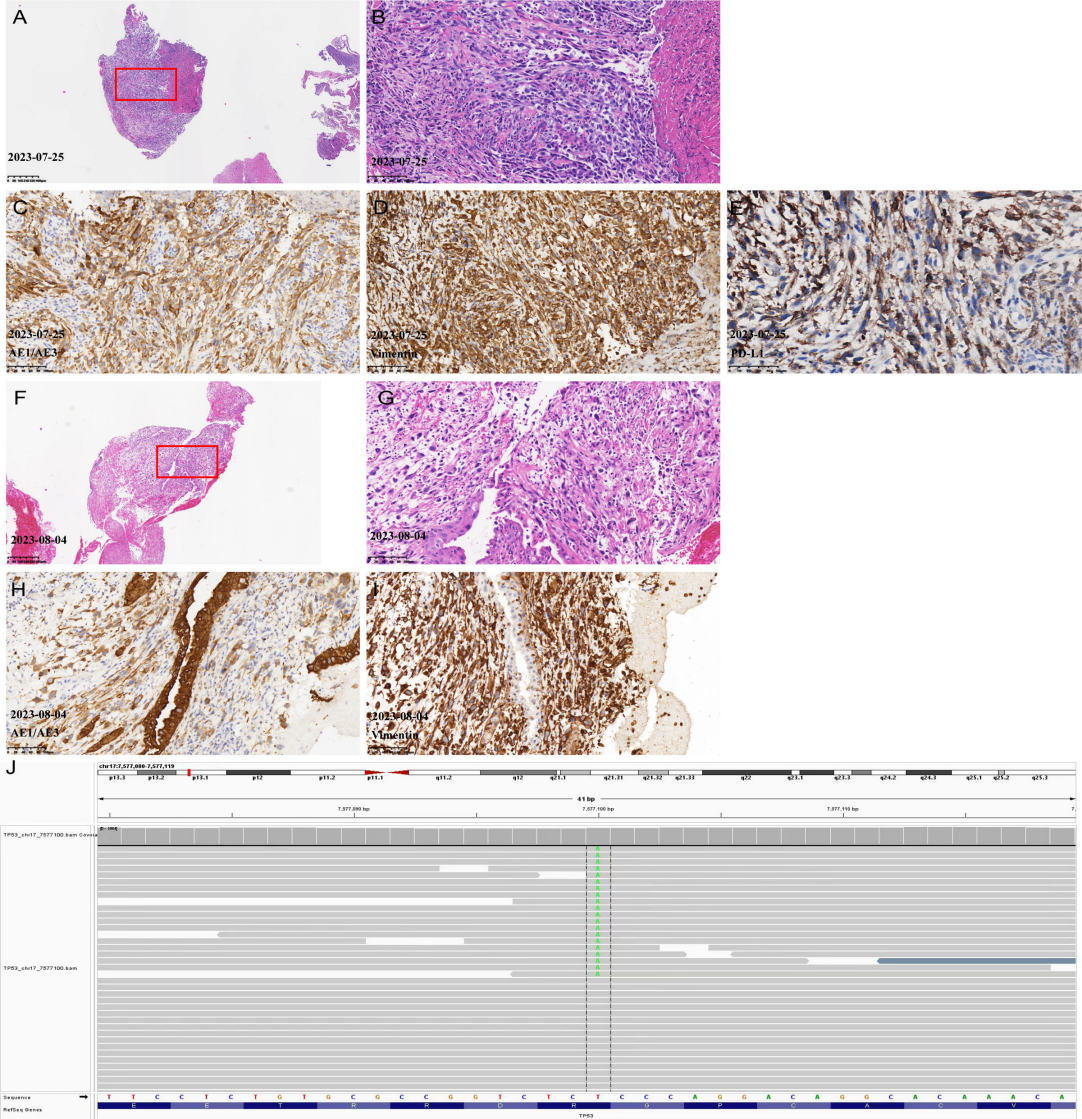
The histopathology and IHC of a biopsy through fiberoptic bronchoscopy confirmed the diagnosis of right upper PSC with necrosis. after the patient received afatinib targeted therapy for 2 months. **(A, B)** H&E stain on July 25th, 2023, **(A)**original magnification ×40, **(B)** original magnification ×200. **(C–E)** Immunohistochemistry of right upper PSC on July 25th, 2023, original magnification ×200, **(C)** IHC AE1/AE3, **(D)** IHC Vimentin. **(E)** IHC PD-L1. **(F, G)** H&E stain on August 04, 2023, **(F)**original magnification ×40, **(G)**original magnification ×200. **(H, I)** Immunohistochemistry of right upper PSC on August 04, 2023, original magnification ×200, **(H)** IHC AE1/AE3, **(I)** IHC Vimentin; **(J)**TP53 mutations based on genetic testing. AE1/AE3, Cytokeratin Antibody to Epidermis1/Antibody to Epidermis3, PD-L1, Programmed Death-Ligand 1, TP53, Tumor Protein p53, IHC, immunohistochemistry.

From 2023 April 20th to 2023 July 9th, the patient was administered with 30 mg of afatinib tablets once a day. From 2023 April 24th to 2023 May 5th, the patient underwent targeted radiation therapy for lumbar vertebrae 5. Chest CT on July 14, 2023 showed a mass-like opacity in the surgical area of the right upper lung, measuring 5.3 cm × 3.8 cm, with heterogeneous enhancement on contrast-enhanced scan. Compared with the baseline CT on April 19, 2023, the lesion was significantly enlarged, and the efficacy evaluation was progressive disease (PD) (**Figure 1A**(d). MRI performed on the same day showed compression and flattening of the L5 vertebral body. Nodular and patchy abnormal signals were observed in the L5 vertebral body and its right posterior element, presenting hypointensity on T1WI, hyperintensity on T2WI and fat-suppressed sequences, with marked heterogeneous enhancement on contrast-enhanced scan. Compared with the T2WI signal on April 19, 2023, the T2WI signal intensity decreased, indicating improvement of the osteolytic metastatic lesion in the L5 vertebral body (**Figure 1B**(b). On July 19, 2023, fiberoptic bronchoscopy-guided biopsy was performed. Histopathological assessment of the biopsy specimen on July 25 confirmed the diagnosis of right upper lobe PSC with necrotic changes. Hematoxylin and eosin (HE) staining revealed predominantly columnar tumor cells, admixed with epithelioid and spindle-shaped (interstitial) subtypes. The tumor cells exhibited marked nuclear and cytoplasmic atypia, arranged in a fascicular growth pattern (**Figures 3A, B**). IHC profiling showed diffuse positivity for the pan-cytokeratin marker AE1/AE3 (**Figure 3C**) and the mesenchymal marker vimentin (**Figure 3D**), consistent with the biphasic differentiation characteristic of PSC. The Ki-67 proliferation index was 60%, indicative of a high tumor proliferative activity and aggressive biological behavior. A repeat video bronchoscopy with biopsy was performed on August 4, 2023, and histopathological evaluation yielded results concordant with the initial diagnosis (**Figures 3F–I**). IHC assessment of programmed death ligand 1 (PD-L1) expression demonstrated a tumor proportion score (TPS) of 85% and a combined positive score (CPS) of 90%, defined as PD-L1 positivity per clinical testing criteria (**Figure 3E**). Gene mutation detection by fluorescence polymerase chain reaction (fluorescence PCR) on August 8, 2023 identified a TP53 mutation with an allele frequency of 9.88% (**Figure 3J**), while no pathogenic alterations were detected in ALK, ROS1, EGFR, MET or RET. Between July 27 and November 10, 2023, the patient received 5 cycles of immunochemotherapy, with the following regimen: sintilimab 200 mg via intravenous infusion (IV infusion) on day 1; albumin-bound paclitaxel 200 mg on day 1 and 100 mg on day 8, administered via IV infusion; carboplatin 450 mg, with a treatment cycle of every 3 weeks (Q3W/C). After 2 cycles of treatment, CT scan on September 10, 2023 revealed irregular osteolytic destruction in the L5 vertebral body and its right posterior element. The L5 vertebral body was flattened, with bone deposition and increased density observed (**Figure 1B**(c)). Chest CT on September 10, 2023 showed significant reduction of the mass from baseline CT (July 14, 2023), achieving a partial response (PR) to the solid tumor response evaluation criteria (**Figure 1A**(e) based on Response Evaluation Criteria in Solid Tumors, which is a standard for evaluating the efficacy of solid tumors. A chest CT on November 26, 2023 showed mass disappearance, and a PET-CT on November 27, 2023 confirmed the same conclusion (**Figure 1A**(f), along with the disappearance of L5 vertebra lesions (**Figure 1B**(d). PET-CT showed that the recurrent lesion in the right upper lung had regressed and become metabolically inactive, with no abnormal soft tissue density shadow observed on CT. The metastatic lesion in the L5 vertebral body and right pedicle also presented significant regression and metabolic inactivation on PET-CT, with a maximum SUV of 1.8. Meanwhile, PET-CT confirmed complete disappearance of metabolic activity in all lesions (all SUV values < 2.0) and no new lesions were detected. Tumor markers including CA125 and ferritin both returned to normal ranges.

## Discussion

With the emergence of targeted therapy, EGFR-tyrosine kinase inhibitors (TKIs) have become a crucial component in the treatment of EGFR-mutated NSCLC, including postoperative adjuvant therapy and advanced palliative care (8). Despite the potency of these inhibitors, resistance is still expected to emerge (14). Globally, erlotinib, afatinib, and osimertinib are among the first-line treatments for NSCLC with EGFR-activating mutations, which have significantly transformed clinical practice (15). To date, several acquired resistance mechanisms have been documented, including secondary EGFR mutations, bypass or alternative pathway activation, and histological transformation (16). This study describes a case of early-stage NSCLC patient with an EGFR gene exon-21 L858R mutation who underwent radical surgery and refused adjuvant therapy. The patient subsequently experienced recurrence and metastasis, and received first-line systemic treatment with afatinib. However, two months later, the patient showed rapid disease progression. Bronchoscopy and pathological biopsy were performed again, and histopathology confirmed that the original right upper lung adenosquamous carcinoma had transformed into sarcomatous carcinoma. Genetic testing revealed that the TP53 mutation had replaced the EGFR mutation, indicating that the transformed PSC sample did not retain the original EGFR mutation, suggesting that the mechanism of resistance to EGFR-TKIs in this case was transformation of the pathological tissue type.

Before surgery, the patient underwent comprehensive staging workup including contrast-enhanced chest CT, bone scan, abdominal ultrasound, and cervical lymph node ultrasound. Distant metastasis was ruled out after MDT discussion, and the postoperative pathological staging was stage IA for the right upper lobe lesion and stage IB for the right lower lobe lesion. However, local recurrence of the right upper lobe lesion and distant L5 vertebral metastasis occurred two weeks after surgery. The patient and her family declined preoperative ctDNA liquid biopsy to evaluate the possibility of occult micro-metastases at initial diagnosis due to financial constraints. Studies have shown that recurrence or metastasis within three months after surgery for NSCLC is uncommon and typically indicates highly aggressive tumor biology, relatively advanced pathological stage, or pre-existing occult micro-metastasis/molecular residual disease (MRD). The MRD positivity rate within 120 days after surgery is approximately 25%, and is significantly associated with an elevated risk of recurrence (17). Multicenter studies have demonstrated that some patients may develop early recurrence within six months after surgery, suggesting that their tumors may lie near the “resectable/unresectable boundary” and exhibit more aggressive biological behavior (18). Common sites of postoperative recurrence include the brain, bone, liver, adrenal glands, and distant lymph nodes; early recurrence is more likely to occur at sites with poor prognosis, such as bone, liver, and kidney (18, 19). Early recurrence is more frequently observed in high-grade histological subtypes (e.g., large cell carcinoma, sarcomatoid carcinoma) and more advanced pathological stages, indicating an inherently rapid progression characteristic (18). Furthermore, it has been proposed that perioperative changes in the immune microenvironment may promote the “awakening” of micro-metastases. A 2024 study in a cohort of 192 postoperative NSCLC patients found that elevated postoperative peripheral blood monocyte count was a risk factor for recurrence; animal models demonstrated that surgical stress rapidly upregulates MCP-1, recruiting monocytes into distant micro-metastatic sites and converting them into pro-tumorigenic tumor-associated macrophages (TAMs), thereby triggering micro-metastatic outgrowth. Inhibition of the MCP-1 axis reduced postoperative distant metastasis (20). A 2025 study in stage III NSCLC proposed that downregulation of the ALCAM-CD6 axis is associated with enhanced immunosuppressive status, cell cycle arrest release, and increased tumor proliferation and migration, suggesting that immune-mediated metastatic recurrence mechanisms may contribute to early relapse (21). Early extensive distant metastasis (“blast metastasis”) is believed to involve complex interactions among host immune surveillance, tumor immune evasion, and tumor virulence factors, rather than necessarily reflecting technical issues with the surgical procedure (22). This clinical course is more consistent with the explanatory framework of “pre-existing dissemination + postoperative microenvironmental changes promoting outgrowth”: on one hand, early metastatic seeding or MRD positivity may have existed prior to surgery; on the other hand, surgical stress-induced recruitment of immunosuppressive cells can accelerate the expansion of micro-metastatic foci (20, 23).

This case highlights the potential of EGFR-TKIs to induce a transformation of the pathological type of lung cancer, possibly from adenocarcinoma to squamous cell carcinoma-like (PSC), which may be an important mechanism of EGFR-TKIs resistance. Another hypothesis for this observation is that the patient had a concomitant histopathological tumor at the initial postoperative pathological diagnosis, which was not evident on the postoperative histopathological samples, and then PSC component became dominant as the adenosquamous carcinoma component was successfully treated with an EGFR-TKIs. However, while it’s still unclear whether this result represents a histopathological transformation or concurrent histopathological tumor, the findings from this case raise several practical questions that could impact clinical practice.

Firstly, in the diagnosis and treatment of cancer patients, including those who are receiving EGFR-TKIs, if the cancer does not respond as originally anticipated, such as rapid progression following treatment with EGFR-TKIs, or inconsistent response to two or more different lesions, is repeat biopsy necessarily required to rule out histologically dominant structures that were not detected at the time of initial diagnosis or to determine whether the histological type of the cancer has shifted? Secondly, NSCLC patients with driver gene mutations who received EGFR-TKIs treatment have shown histological transformation. Is it necessary to further enhance genetic testing in time to identify genomic changes? Lastly, is repeat multipoint biopsy necessarily needed when we have doubts about the histopathological type?

Previous research has shown that SCLC transformation is one of the mechanisms of EGFR-TKIs resistance, accounting for 3% to 14% of all cases (15). And several studies have confirmed that LUAD with EGFR-mutations can transform into squamous cell carcinoma after EGFR-TKI treatment, which guides clinical practice and results in long-term clinical benefits for patients (24). Other studies have reported that lung adenocarcinoma can transform into neuroendocrine tumors (25), squamous cell carcinomas, and large cell neuroendocrine carcinomas as resistance mechanisms of EGFR-TKIs therapy (26). Large cell neuroendocrine carcinoma (27) and squamous cell carcinoma (28) transformation and EGFR-T790M mutation as coexisting mechanisms of EGFR-TKIs in lung cancer, respectively. Interestingly, one case report showed that a lung adenocarcinoma patient developed small cell lung cancer transformation and metastasis to the breast after EGFR-TKIs treatment (29). Additionally, Min-Shu Hsieh et al. found that EGFR-TKIs promoted lung sarcoid transformation by inducing abnormal MET activation, as a mechanism of EGFR-TKIs resistance with a poor prognosis (30).

Both the adenosquamous carcinoma of the right upper lung lobe and the adenocarcinoma of the right lower lung lobe surgically resected in February 2023 harbored the EGFR exon 21 L858R mutation, confirming that the two primary lesions originated from the same EGFR-mutant clone, which is consistent with the characteristics of multifocal clonal dissemination in early-stage lung cancer. The patient refused adjuvant targeted therapy after surgery, and developed lumbar spine metastasis 2 weeks post-operation. After 3 months of afatinib treatment, the pulmonary lesions progressed, and biopsy at this time confirmed sarcomatoid carcinoma. This clinical course fully aligns with the clonal selection mechanism of EGFR-TKI therapy: afatinib effectively eliminated the adenocarcinoma cell population dependent on EGFR signaling, but enriched the pre-existing drug-resistant subclones that carried EGFR mutations and simultaneously exhibited epithelial-mesenchymal transition (EMT) features. Under therapeutic pressure, these subclones underwent phenotypic transformation, lost EGFR expression and acquired sarcomatoid characteristics. Loss of EGFR mutant alleles is a common drug resistance mechanism under targeted therapy pressure. EGFR-driven clones can lose the mutant EGFR allele through chromosomal instability under TKI selection pressure, thus completely escaping dependence on EGFR signaling (31, 32). This phenomenon itself is direct evidence of clonal evolution, rather than a marker of tumors of distinct origins. On the other hand, the lineage transformation process was confirmed at the protein expression level, excluding the possibility of primary pulmonary sarcoma or metastatic tumors from other sites. The epithelial markers (AE1/AE3, CAM5.2) of the initial adenosquamous carcinoma showed diffuse positive expression, and the recurrent sarcomatoid carcinoma also retained positive AE1/AE3 expression, while presenting diffuse positive Vimentin expression simultaneously. This “co-expression of epithelial and mesenchymal markers” phenotype is a typical immunohistochemical feature of sarcomatoid carcinoma, which directly confirms that it originates from epithelial neoplasm and acquires sarcomatoid morphology through epithelial-mesenchymal transition (EMT), rather than being a sarcoma derived from primary mesenchymal tissue. In the initial tumor, the adenocarcinoma component was positive for TTF-1 and the squamous cell carcinoma component was positive for p40, while the expression of the above lineage markers was lost in the recurrent sarcomatoid carcinoma. This phenomenon is highly consistent with epigenetic reprogramming during histological transformation. After EGFR-mutant lung cancer transforms into sarcomatoid carcinoma, the original lineage-specific differentiation genes (such as TTF-1, Napsin A, p40, etc.) are usually silenced (6), while mesenchymal-related transcriptional programs are activated. This histological transformation has been verified in multiple transformation cohorts (33, 34). After the diagnosis of sarcomatoid carcinoma, the patient received immunochemotherapy (sintilimab + albumin-bound paclitaxel + carboplatin). Partial response (PR) was achieved after 2 cycles of treatment, and the lesions completely resolved after 5 cycles, with a PD-L1 TPS as high as 85%. This high PD-L1 expression feature is consistent with the characteristic of elevated immunogenicity of EGFR-mutant lung cancer after transformation, and the clinical response pattern conforms to the sensitivity feature of transformed sarcomatoid carcinoma to immunochemotherapy, which further supports its origin from the initial lung cancer clone. A cohort study on sarcomatoid transformation of EGFR-mutant lung cancer published in 2025 enrolled 10 patients with similar transformation cases, suggesting that the transformation may occur via epithelial-mesenchymal transition (EMT), and all post-transformation tumors shared driver mutations with the primary tumors (35). A study including 81 cases of pulmonary sarcomatoid carcinoma (PSC) found that EGFR mutations were present in 23% of the cases, and KRAS mutations in 46% of the cases (36). A case report showed that histological transformation to sarcomatoid carcinoma may occur after development of resistance to ALK or EGFR targeted therapy, accompanied by molecular alterations such as MET amplification or TP53 mutation (37). Although the existing multi-dimensional evidence is sufficient to support the core conclusion of common clonal origin, due to the absence of whole-exome sequencing or large-panel NGS, we cannot accurately identify all co-mutations at the whole-genome level, construct a clonal evolutionary tree to quantitatively analyze the evolutionary relationship, nor can we completely rule out the theoretically extremely low probability of metachronous double primary tumors. There is also a certain limitation in the depth of explanation of the specific molecular mechanisms underlying EMT and upregulation of PD-L1 expression during the transformation process. Nevertheless, the combined analysis integrating molecular characteristics, immunophenotype and clinical course is adequate to exclude the possibility of double primary tumors.

Lung adenocarcinoma (especially those harboring EGFR L858R mutation) can undergo histological transformation to PSC under the selective pressure of EGFR-TKIs, which is essentially a lineage reprogramming process driven by tumor cell plasticity. Among the underlying mechanisms, YAP/TAZ-mediated Hippo pathway dysregulation and the ZEB1-centered epithelial-mesenchymal transition (EMT) program have been repeatedly identified as the key molecular axes driving the “epithelial-mesenchymal/sarcomatoid” phenotypic switch, and are coupled with phenotypes of drug resistance, invasive metastasis, and immune evasion (38–40). Under targeted therapy pressure, tumors can achieve drug resistance through non-hereditary cell state switching. In addition to neuroendocrine transformation, transdifferentiation of adenocarcinoma to squamous cell carcinoma, sarcomatoid carcinoma and other subtypes are also classified into this resistance paradigm (39, 41). After targeted inhibition, a slowly proliferating cell population may emerge, whose phenotype can maintain a certain proliferative capacity and acquire further evolutionary potential subsequently. This process is often accompanied by epigenetic remodeling and chromatin state changes, which constitute the basis of “reversibility/plasticity” (41, 42). PSC transformation may proceed via a multi-step, parallel evolution pattern involving multiple subclones. Previous case reports have shown that the same patient can develop squamous cell carcinoma, PSC, and even neuroendocrine components sequentially at different sites, with distinct acquired alterations detected at different stages, suggesting dynamic reorganization of “trunk clones + branch clones” under drug selection (43). When the Hippo pathway is inactivated, YAP/TAZ are dephosphorylated and translocate into the nucleus, driving transcriptional programs related to proliferation, migration, and survival. Dysregulation of this axis is associated with chemoresistance, stemness, immune evasion, and other malignant phenotypes (38). One *in vitro* study demonstrated that EGFR activation (overexpression/amplification/mutation) can phosphorylate MOB1 and inactivate LATS1/2, thereby activating YAP/TAZ independently of traditional upstream Hippo kinases. A positive feedback loop between EGFR-ligand-activated YAP also exists, suggesting that YAP/TAZ may act as an “amplifier” to promote lineage remodeling and tumor progression in the context of EGFR driver alterations (43). Abnormal YAP1/TAZ activation can upregulate EMT-inducing transcription factors (including SLUG and ZEB1), promote E-cadherin downregulation and enhance invasiveness; meanwhile, it can upregulate stemness-related factors such as SOX2 and OCT4, strengthening the potential for recurrence and drug resistance (38). Multiple studies have interpreted PSC transformation as an EMT process. After transformation, typical EMT features such as vimentin positivity, reduced expression of E-cadherin and epithelial markers can be observed, which is considered a potential therapeutic target (43, 44). ZEB1, a key regulatory node of EMT, can bind to the E-cadherin promoter and inhibit its expression. Elevated ZEB1 expression in lung cancer cells is closely associated with EMT, enhanced migration, and invasiveness (45). In the scenario where EGFR L858R-mutant lung adenocarcinoma undergoes pulmonary sarcomatoid carcinoma (PSC) transformation after EGFR-TKI treatment accompanied by EGFR L858R off-target/loss, the core role of TP53 mutation lies in abrogating the tumor suppressor function of wild-type p53, inducing genomic instability and transcriptional reprogramming, thus accelerating resistance evolution and driving the epithelial-mesenchymal/sarcomatoid phenotypic transition. Meanwhile, TP53 mutations are frequently co-enriched with RB1 deletion, MET aberrations, and PI3K pathway activation, which collectively constitute the molecular basis of PSC transformation and are associated with poorer prognosis and more rapid disease progression (46–48). TP53 mutation is one of the most common concurrent mutations in EGFR-mutant non-small cell lung cancer (NSCLC), and is correlated with shorter progression-free survival (PFS) and overall survival (OS). Its main impact is not reducing the depth of initial response, but accelerating the acquisition of resistance mechanisms, and this effect exists independently of specific resistance mechanisms (e.g., T790M) (46–48). Sarcomatoid transformation can occur via EMT and is associated with adverse outcomes; meanwhile, it is emphasized that tissue biopsy should be performed at the progressive stage to evaluate transformation and resistance mechanisms (49). Although sarcomatoid transformation is rare (<5%), it serves as a shared resistance pattern across targeted therapies for diverse driver alterations. Post-transformation samples usually retain the founding mutation, accompanied by high-frequency alterations of TP53, RB1, MET and PI3K activation signals, suggesting that TP53 mutation is an important cooperating event in PSC development (47). Mutant TP53 can form a transcriptional complex with HIF1α at the promoter region of DSG3, upregulate DSG3 in a gain-of-function (GOF) manner and promote invasive phenotype via the Ezrin pathway, providing a mechanistic explanation for the high invasiveness of the “sarcomatoid/mesenchymal-like” phenotype (50). In TP53-mutant LUAD, enriched spatially correlated interactions such as CD274/PDCD1 and PVR/TIGIT can be observed, accompanied by a pro-metastatic niche composed of SPP1^+^ macrophages and collagen-expressing fibroblasts, suggesting that TP53 mutation not only alters the intrinsic programs of tumor cells, but also shapes a tumor microenvironment with stronger immunosuppressive propensity (51). The frequency of TP53 mutations is very high in PSC, and TP53 mutations are associated with an inflammatory immune phenotype, high PD-L1 expression and higher TMB to some extent. Therefore, after PSC transformation, immunotherapy (including combination with anti-angiogenic agents) may become one of the important treatment directions (52, 53). The biological basis for the relatively more favorable response of pulmonary sarcomatoid carcinoma (PSC) to immune checkpoint inhibitors (ICIs) is mainly derived from its extremely high proportion of programmed death-ligand 1 (PD-L1) overexpression. Multicenter retrospective studies have shown that PSC achieves better outcomes with ICI treatment than chemotherapy, and this improvement is predominantly driven by high PD-L1 expression (≥50% in 73%–77% of cases). In populations with high PD-L1 expression, response rates are comparable across different histological subtypes, suggesting that the immunotherapeutic benefit in PSC depends more on PD-L1 status rather than histology itself (54). STK11 mutation/deletion leads to an immunosuppressive tumor microenvironment (TME), characterized by reduced CD8^+^ T cell infiltration, increased expression of T cell exhaustion markers, and accumulation of tumor-associated neutrophils, which is associated with non-response to ICIs (55). Most PSC tumors exhibit an immune-inflamed phenotype, commonly characterized by a type I adaptive immune resistance pattern (PD-L1^+^/CD8^+^), which constitutes an important biological basis for the sensitivity of PSC to immunotherapy (56). Comprehensive analysis of PSC surgical specimens has demonstrated that PSC harbors higher immune cell infiltration compared with conventional NSCLC, including increased CD3^+^ tumor-infiltrating lymphocytes (TILs) and CD163^+^ tumor-associated macrophages (TAMs); moreover, increased infiltration of CD3^+^ and CD4^+^ TILs is associated with longer overall survival (OS) (56). The PD-L1 positivity rate in PSC is significantly higher than that in common NSCLC subtypes. In a Chinese PSC cohort, the PD-L1 positivity rate was 80%, of which 60% exhibited high PD-L1 expression (TPS ≥ 50%) (57). Multicenter retrospective studies have shown that TMB in PSC is comparable to that in LUAD and lung squamous cell carcinoma (LUSC), and is not significantly correlated with ICI outcomes; the ICI benefit in PSC is primarily driven by high PD-L1 expression (54). No correlation exists between PD-L1 expression level and relative TMB; therefore, TMB should not be used as a substitute for PD-L1 in predicting ICI benefit in PSC (58). Although some studies have reported high TMB in PSC, multicenter data suggest that its discriminatory power for ICI outcomes is limited, and clinical decision-making should rely more on PD-L1 status for stratification (54, 58). A Chinese PSC cohort demonstrated that ICI application is independently associated with better prognosis. Among patients receiving ICI treatment, the objective response rate (ORR) was approximately 34.5%, median progression-free survival (mPFS) was approximately 12.5 months, and median overall survival (mOS) was approximately 16.0 months, collectively suggesting that PSC has a strong biological basis for benefiting from immunotherapy (57). In STK11-mutant lung adenocarcinoma (LUAD), tumor-derived complement component 3 (C3) upregulation is correlated with poorer survival. STK11 knockout increases neutrophil recruitment, reduces T cell infiltration, and confers resistance to anti-PD-1 therapy, while C3 deletion restores anti-PD-1 sensitivity, indicating that STK11 aberrations enhance immune escape via the complement axis (59). STK11/LKB1-mutant non-small cell lung cancer (NSCLC) lacks TCF1^+^ CD8^+^ T cells, which is associated with ICI refractoriness. AXL inhibition promotes type I interferon secretion by dendritic cells and expands TCF1^+^ PD-1^+^ CD8^+^ T cells, thereby restoring the efficacy of PD-1 blockade, and clinical responses to such combination regimens have been observed (60). Studies have found that despite the overall high PD-L1 expression in PSC, some patients with high PD-L1 expression still develop hyperprogressive disease (HPD), and it has been proposed that HPD may be associated with STK11 mutations. Other studies have also shown a correlation signal between HPD and STK11 mutations in NSCLC (53). Given the relatively low prevalence of STK11 alterations in PSC, STK11 abnormalities are more appropriately considered as supplementary information for potential reduced ICI benefit/increased HPD risk. When STK11 aberrations are present, avoiding ICI monotherapy and prioritizing combination strategies should be considered, with attention to research progress on actionable targets such as AXL (53, 54, 60). Therefore, STK11 testing is recommended for risk stratification rather than treatment exclusion.

Jinyang Zheng et al. found that pulmonary sarcomatoid transformation is a relatively rare mechanism of EGFR-TKIs resistance, and gefitinib may promote this transformation by alternately amplifying MET and EGFR T790M mutation. At the same time, they observed high expression of MET, numerous MET copies, and high PD-L1 expression (61). Compared to conventional NSCLC, PSC has the ability to metastasize to unusual sites such as the kidney, pancreas, skin, and digestive tract. Thus, PSC transformation might be associated with MET amplification, PD-L1 overexpression, and metastasis to abnormal sites. Additionally, another study found that an aggressive LUAD patient with ALK rearrangement converted to PSC after osimertinib treatment, which became an important new resistance mechanism for EGFR-TKIs (37). Interestingly, a previous study reported that aggressive LUAD with EGFR-mutation first transformed into squamous cell carcinoma and then into lung sarcomatoid carcinoma after treatment with an EGFR-TKI (62). Our study found that histological transformation of lung adenosquamous carcinoma with EGFR Exon-21 L858R mutation into PSC after patients treated with afatinib was a novel and important resistance mechanism of EGFR-TKIs. Even more wonderful, in our case, lung adenosquamous carcinoma harboring EGFR Exon-21 L858R mutation transformed into lung sarcoma carcinoma after treatment with afatinib. Subsequently, gene expression analysis revealed a TP53 mutation and high PD-L1 expression. Primary lesion disappeared after the patient received albumin-bound paclitaxel combined with carboplatin chemotherapy, and immunotherapy with sindiglizumab. The patient achieved a durable complete response to sintilimab-based immunochemotherapy, with no recurrence during the 4-month follow-up, which is significantly superior to the survival outcomes reported in previous literature. This case not only enriches the phenotypic spectrum of EGFR-TKI resistance-related histological transformation, but also provides clinical evidence that immunochemotherapy can achieve long-term disease control in patients with sarcomatoid transformation and high PD-L1 expression. Postoperative dynamic circulating tumor DNA (ctDNA) testing can provide direct evidence-based evidence for the processes of clonal clearance and clonal transformation during targeted therapy (63–65). However, this study is a retrospective case report: no plasma samples were prospectively collected during the patient’s postoperative follow-up, and no retained plasma samples are currently available for ctDNA detection, which is an inherent limitation of retrospective study design. Future studies should prospectively incorporate dynamic ctDNA monitoring to further elucidate the clonal evolution mechanism underlying histological transformation.

## Data Availability

The raw data supporting the conclusions of this article will be made available by the authors, without undue reservation.
