# Supplementary material for: Histological transformation into pulmonary sarcomatoid carcinoma from lung adenosquamous carcinoma after radical resection of EGFR gene Exon-21 L858R mutation: a case report and literature review
Source: Front Oncol. 2026 Jun 18;16:1811812. doi: 10.3389/fonc.2026.1811812 (PMC13322830; doi:10.3389/fonc.2026.1811812)
Supplement: Supplementary file 1 [file DataSheet1.docx]

Supplementary Figure


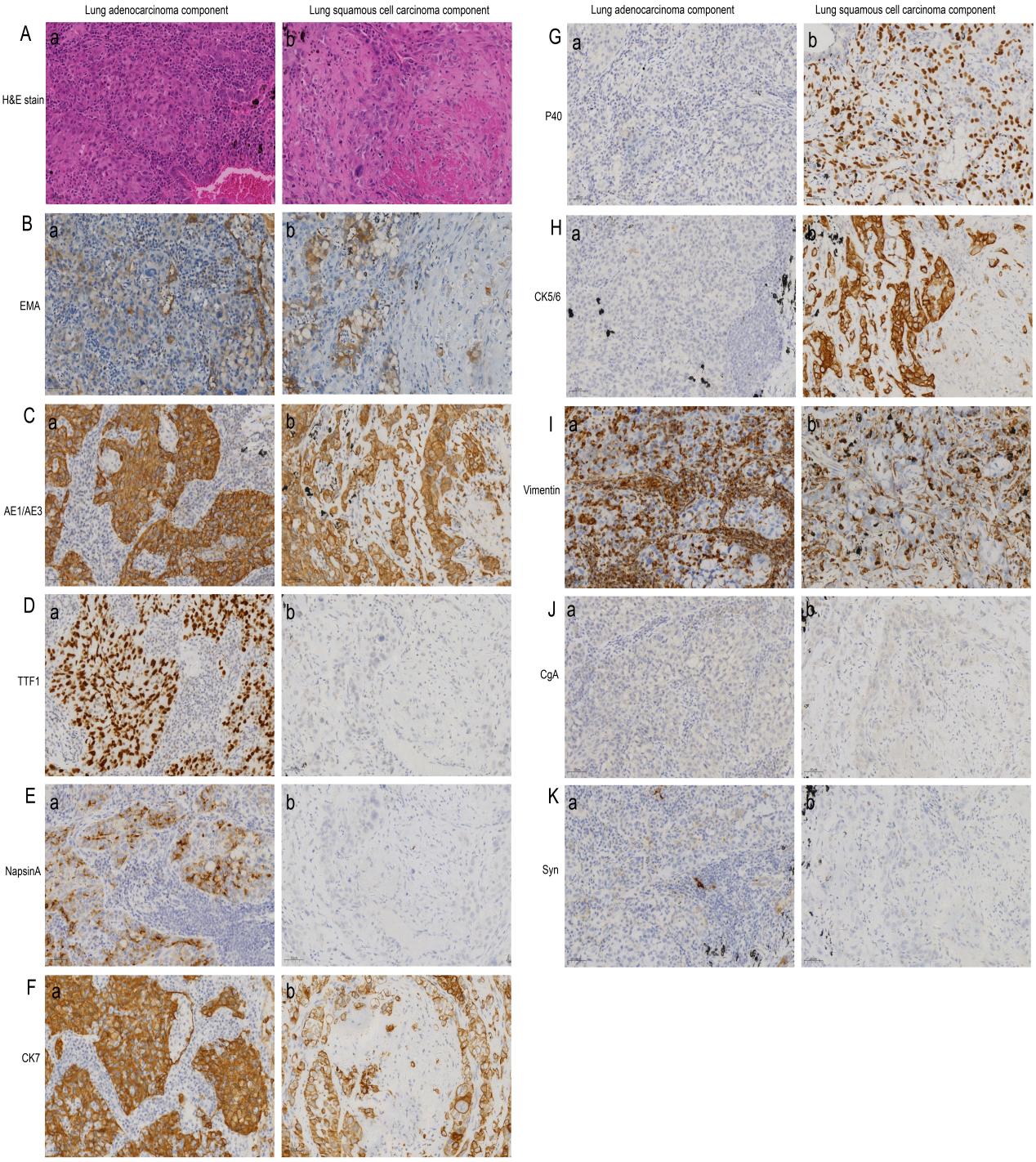


**Supplementary Figure** Right upper lung nodule for immunohistochemical detection. (A) HE staining of adenocarcinoma(a) and squamous cell carcinoma(b) components. (B-K) The EMA, AE1/AE3, TTF1, NapsinA, CK7, P40, CK5/6, Vimentin, CgA and Syn immunohistochemical staining in lung adenocarcinoma(a) and squamous cell carcinoma(b) components. Epithelial Membrane Antigen=EMA, Anti-Cytokeratin1/3=AE1/3, Thyroid Transcription Factor 1=TTF1, Cytokeratin 7=CK7, Delta N isoform of p63=ΔNp63=P40, Cytokeratin 5/6=CK5/6, Chromogranin A =CgA, Synaptophysin =Syn.
